# Behavioral mechanisms and morphological symptoms of zombie ants dying from fungal infection

**DOI:** 10.1186/1472-6785-11-13

**Published:** 2011-05-09

**Authors:** David P Hughes, Sandra B Andersen, Nigel L Hywel-Jones, Winanda Himaman, Johan Billen, Jacobus J Boomsma

**Affiliations:** 1 Departments of Entomology and Biology, Penn State University, PA 16802, USA; 2 Centre for Social Evolution, Department of Biology, University of Copenhagen, Universitetsparken 15, 2100 Copenhagen, Denmark; 3 Mycology Laboratory, National Center for Genetic Engineering and Biotechnology, Science Park, Pathum Thani 12120, Thailand; 4 Forest Entomology and Microbiology Group, National Park, Department of National Parks, Wildlife and Plant Conservation, Bangkok 10900, Thailand; 5 Zoological Institute, University of Leuven, Naamsestraat 59, 3000 Leuven, Belgium

**Keywords:** extended phenotype, behavioral manipulation, ants, fungi, convergent evolution, parasites

## Abstract

**Background:**

Parasites that manipulate host behavior can provide prominent examples of extended phenotypes: parasite genomes controlling host behavior. Here we focus on one of the most dramatic examples of behavioral manipulation, the death grip of ants infected by *Ophiocordyceps *fungi. We studied the interaction between *O. unilateralis s.l*. and its host ant *Camponotus leonardi *in a Thai rainforest, where infected ants descend from their canopy nests down to understory vegetation to bite into abaxial leaf veins before dying. Host mortality is concentrated in patches (graveyards) where ants die on sapling leaves *ca*. 25 cm above the soil surface where conditions for parasite development are optimal. Here we address whether the sequence of ant behaviors leading to the final death grip can also be interpreted as parasite adaptations and describe some of the morphological changes inside the heads of infected workers that mediate the expression of the death grip phenotype.

**Results:**

We found that infected ants behave as zombies and display predictable stereotypical behaviors of random rather than directional walking, and of repeated convulsions that make them fall down and thus precludes returning to the canopy. Transitions from erratic wandering to death grips on a leaf vein were abrupt and synchronized around solar noon. We show that the mandibles of ants penetrate deeply into vein tissue and that this is accompanied by extensive atrophy of the mandibular muscles. This lock-jaw means the ant will remain attached to the leaf after death. We further present histological data to show that a high density of single celled stages of the parasite within the head capsule of dying ants are likely to be responsible for this muscular atrophy.

**Conclusions:**

Extended phenotypes in ants induced by fungal infections are a complex example of behavioral manipulation requiring coordinated changes of host behavior and morphology. Future work should address the genetic basis of such extended phenotypes.

## Background

Some parasites can adaptively take over and completely control the behavior of their hosts leading to positive fitness returns for parasite genes [1-4]. Host behavior is an extended phenotype of the parasite [5]. The degree of behavioral manipulation varies greatly across parasites from very slight alterations of pre-existing behaviors [6] to the expression of wholly novel behaviors that are never seen in healthy hosts [7]. Extended phenotypes have gained considerable prominence in community- [8], evolutionary- [9] and behavioral ecology [10].

Early studies of extended phenotypes focused on detailing behavioral changes and inferring whether they represent adaptations for parasites or should rather be interpreted as adaptive defense mechanisms of the host or as by-products of infection [11-13]. Recently, more integrative approaches have emerged which includes a greater focus on the mechanisms by which behavioral changes occur. An important component is a fuller understanding of the biology of particular study systems and the timing of observation or experimentation, since parasite induced behavioral changes are highly dynamic [14].

Here we focus on a study system that is a dramatic example of adaptive manipulation of animal behavior by a parasite. Worker ants infected by fungal parasites belonging to the genus *Ophiocordyceps *express death grip behavior shortly before dying for no apparent other purpose than to assist parasite reproduction [15,16]. Worker ants are infected during foraging by spores that attach to the cuticle. The fungus is an obligate, directly transmitted parasite that requires ants for reproduction. Germination and subsequent penetration of the cuticle lead to rapidly progressing infections inside the host body [17,18], but fungal reproduction is only possible after the growth of a large stalk from the back of the ant's head followed by a propulsive release of spores from this fruiting body [16]. The fungus inevitably kills the ant and must do this outside the colony because ants quickly remove dead nest-mates [19], so that dying in the nest would not allow sufficient time for stalk development and spore release [20]. This host death as a developmental necessity implies that *Ophiocordyceps *infections would also match the functional definition of being a parasitoid [21].

While the *Ophiocordyceps *clade has a global distribution local interactions tend to be highly specific with highly stereotyped host behaviors [16,22]. Ants of the tribe Camponotinii (*Camponotus, Polyrhachis *and *Echinopla*) are known to leave their nest to bite into leaves before dying from infections with a representative of the species complex *Ophiocordyceps unilateralis sensu lato *[16]. (See taxonomic note in Methods). A recent study in a Thai rainforest showed that leaf biting behavior by infected workers of this ant species was adaptive for the fungus because it secures a stable microclimatic niche for the *post mortem *development of the stalk and the subsequent release of spores [20]. In this intensively studied population infected worker ants leave their colony in the dry, hot canopy and descend to the humid understory where they appear to actively select leaves of saplings *ca*. 25 +/- 3 cm above the soil surface [20]. These parasite manipulated ants always bite into abaxial leaf veins and not the laminar blade, edge or upper surface (adaxial). They also predominantly die in areas where the cadavers of previously manipulated ants are already abundant leading to graveyards where local densities of ants killed by the fungus may exceed 25/m^2 ^[23]. Graveyards of *Ophiocordyceps *infected ants have also been reported from other continents [24,25]. The healthy ants, though nesting in the high canopy, do periodically walk in the understory leading to new infections [23].

Behavioral manipulation of worker ants by these fungi creates zombie ants [2,4]. Once infected ants exit their colony to die they have no further fitness gains through their own actions (being sterile workers they only have inclusive fitness via helping nest-mates). In fact, by dying within the foraging area of their own colony their behavior may still reduce inclusive fitness [26-28]. The term zombie ants underlines that, while the manipulated individual may look like an ant, it represents a fungal genome expressing fungal behavior through the body of an ant.

Using the explicit 'parasite's eye view' framework outlined above, we set out to test two hypotheses. First, we hypothesized that pre-biting behavior may have an important function to help positioning dying ants in death biting habitats that would be optimal for subsequent fungal reproduction. Second, we hypothesized that the death grip requires changes in the mandibular muscles to transform functional mandibles into death grip lock-jaws to secure that dead ants become permanently fixed to leaves against the force of gravity.

## Results

### Pre-biting behavior

**Figure 1:**
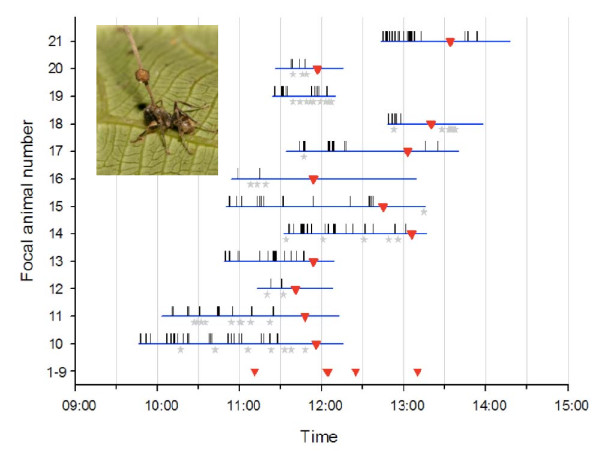
**Zombie ant behavior**. Focal animal observation periodogram of ants infected by *Ophiocordyceps unilateralis s.l*. The blue horizontal bars mark the observation period, the red triangles mark moment of biting, the vertical bars mark spasm events and the grey diamonds the falling off events. For four individuals that belong to focal animals 1-9 only the biting time was recorded. The biting time was recorded for 16 ants but only 15 triangles are visible as two ants bite at exactly the same time (12:05). Inset picture shows a dead ant on a leaf with the fungal stalk and spore body that emerged from the head.

**Figure 2:**
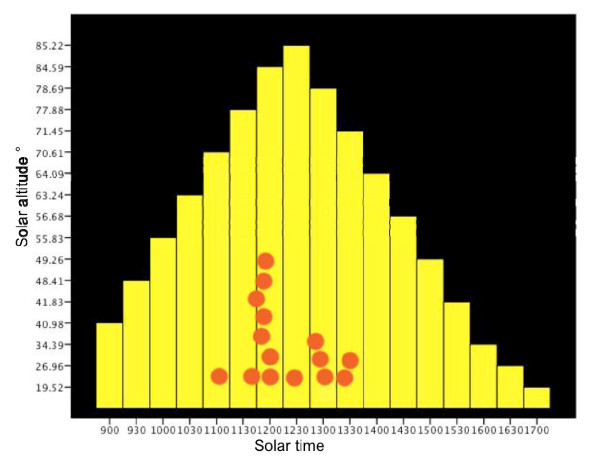
**Synchronized manipulation of ants by fungi**. A sun position chart of the death grip. Solar altitude is represented by the yellow bars and plotted against the y-axis and the biting times are the red circles and plotted on the x-axis in solar time (this is true local time accounting for longitude and different from Time in Figure 1). The red circles are stacked to prevent overlapping. At 11:47 two ants bite so only 15 circles are visible though 16 ants were recorded.

**Figure 3:**
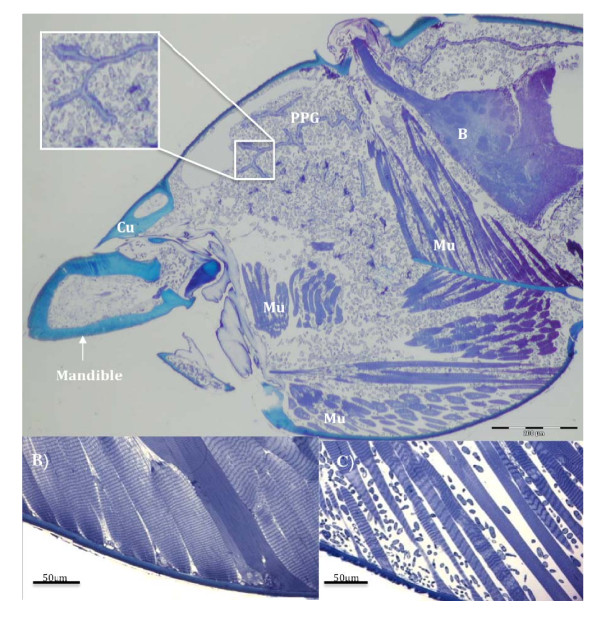
**Heads of manipulated ants colonized by fungi**. A (top panel) is a light micrograph (LM) saggital section through the head of an *O. unilateralis s.l *infected ant that was biting a leaf at the moment of fixation (i.e. alive). The small grey blobs are fungal hyphal bodies that fill the head and mandibles. Note the spacing between the muscle fibers. The insect shows a close up of hyphal bodies around the post-pharyngeal gland (PPG). B is the brain, Mu, Muscles and Cu is cuticle. B) is a LM of healthy muscle and C) is a LM of muscle from a behaviorally manipulated ant that was biting a leaf and alive when removed for fixation. The small blobs between the fibers are fungal cells.

The ant species that is the primary host of *Ophiocordyceps unilateralis s.l*. at our field site is *Camponotus leonardi *[23]. This ant is canopy dwelling, rarely descending to the forest floor and when it does it always travels on well defined trails (Additional File 1). Trail individuals do not forage on the forest floor and trails normally ascend into the canopy within 3-5 m from where they descended (suggesting that workers descend only because breaks in the canopy necessitate a descent to reach adjacent foraging crowns in the canopy). Unlike ants on trails the manipulated ants in the pre-leaf biting stage were all discovered walking alone on low vegetation, usually on saplings <50 cm above soil level and only during the time interval 09:30-12:45 h (n = 21, Figure 1 and 2). All 21 zombie ants that we followed were confirmed to be infected either via dissection of the head to reveal fungal cells or by observing the emergence of *O. unilateralis s.l*. following death on the leaf (Figure 3a). Post mortem fungal growth starts with abundant hyphae emanating from the intersegmental membranes within 2-3 days after host death and ultimately leads to stalk formation from the back of the ant's head [20].

The host ant is diurnal at our field site [23] and infected ants (n = 42) appeared even more restricted in their activity as they were never observed in the early morning or late afternoon (15:00-18:00 hrs), in spite of our searches covering these early and late periods of the day. The understory vegetation of our study site was extensively searched during a year-long census program that examined every leaf below 2 m height in 1360 m^2 ^of forest habitat [23]. We therefore conclude that pre-biting infected *C. leonardi *ants at this site were only active in the morning and that this observation was not affected by sampling bias. The occasional trails of healthy ants that can be found on the forest floor (Additional File 1) were observed both during the morning hours and in the late afternoon, with activity on trails always ceasing around sunset, i.e. between 17:00-18:00 h.

Since behavioral manipulation alters normal behavior we could not *a priori *exclude the possibility that infected individuals would have become nocturnal. We therefore conducted evening (after 18:00 h) and night (22:00-0:00 h) surveys using torches, but did not find any *C. leonardi *ants active in the dark. Furthermore, a collected colony of *C. leonardi *that likely contained some naturally infected ants was maintained under field laboratory conditions for 2 days and did not show any activity in the dark, suggesting that behaviorally manipulated *C. leonardi *ants remain only active during daylight.

We also performed 20 hours and 28 minutes of focal observations on 12 infected ants that were found walking alone (infection status was later confirmed as described above)(see Figure 1, individuals 10-21). These individuals all expressed irregularly spaced whole body convulsions (vertical bars on the periodogram in Figure 1), which often made the ant fall from the vegetation onto the ground (denoted as stars on the periodogram). After falling infected ants always resumed walking and always climbed a small sapling or comparable plant, which were abundantly present in the understory.

We never observed trail ants falling from vegetation. To document this, we removed 13 such ants from a trail on a liana approximately 1 m above the forest floor in the same area where we observed the behaviorally manipulated ants. The liana descended from the canopy and the trail ascended into the canopy via a tree trunk less than 3 m from where we collected the ants. The trail ants (assumed to be uninfected) were placed on the ground and they all quickly ascended into the canopy where we collected them again from tree trunks *ca*. 1.5 m above ground. The only exception was one trail ant that was predated upon by a spider (none of the behaviorally manipulated ants we observed were predated upon). Trail ants did not spend extensive time walking in the understory. Their median time between release and reaching the trunk on which they ascended into the canopy was 28 minutes (range 7-51, total observation time 6 h, 2 m). After these observations, the collected trail ants were maintained singly without food and died within a few days without signs of *O. unilateralis s.l*. fungal growth.

Before biting a leaf, infected ants were predominantly walking (average proportion of time walking 0.62, range 0.11-1.00, total observation time 15 h, 35 m). They traversed an average of 99 leaves (range 52-239, 8 focal ants), which was *ca*. twice the number traversed by trail ants (average 51, range 8-140, 13 focal ants). Because trail ants were never observed walking on leaves except at the times when we removed them from trails and placed them on the ground we conclude that traversing leaves is not a normal behavior. Therefore we did not statistically test for a difference between the numbers of leaves traversed, as this was not biologically meaningful.

During the pre-biting phase behaviorally manipulated ants appeared to express a random "drunkard's walk" such that an individual remained close to its starting point [29] but precise trajectories were not mapped so this remains a heuristic assessment. In all cases the infected ants finally bit into leaves <3 m from where they were first observed.

The timing at which infected ants bit into leaves was synchronised around noon (Figure 1 &2; n = 16), suggesting either a direct solar cue or an indirect one via correlated temperature or humidity. The solar elevation at the moment of biting was 80.28° +/- 1.32 SE, which was close to the maximum solar elevation of 87.29° +/- 0.39 SE during our study period. Once they had bitten leaves, ants rarely became detached and when this happened it was due to disturbance (two cases, # 15 and 18 in Figure 1, after very heavy rain). Biting leaves is not part of the repertoire of healthy ants of this species.

### Post-death grip behavior

After biting into leaves infected ants always died as this is a developmental necessity for the subsequent growth of the fungus [15,20]. It was not possible to determine the exact time of death since obvious signs such as muscle activity could be the result of fungal action, but it did appear that ants could remain alive for as long as six hours after biting. Video recordings of six live ants biting leaves revealed very little behavior of interest besides a periodic twitching of the legs (Additional File 2). The arrival of an ant of a different species close to a biting ant provoked no responses (Additional File 2), in contrast to healthy ants on trails, which were very aggressive to other ant species they encountered at food sources as well as to flying insects like wasps and flies that landed near honey baits.

### Muscular atrophy accompanies behavioral manipulation

At the moment of the death grip, when the ant is under fungal control and biting into the major vein of a leaf its head is filled with fungal cells (Figure 3). These cells, called hyphal bodies, were very abundant and could be found between the muscle fibers and surrounding the brain and post pharyngeal gland (Figure 3), but not inside muscles, brains or glands.

The most prominent other sign of infection, besides the abundance of fungal cells inside the head capsule, was that the mandibular muscles were atrophied. We sectioned the heads of 10 ants that were biting leaves and the pathology was the same across all 10. Mandibular muscle fibers, which normally attach to the head capsule, often appeared to have become detached (Figure 3c) and where fibers remained attached they were stretched (compare 3b and 3c). Ant workers have both mandibular opening and closing muscles and these can be discriminated in healthy ants by their typical length of sarcomeres: 2-3 μm for opening muscles and 5-6 μm for closing muscles (Figure 3b). However, in parasitized ants the characteristic stretching of sarcomeres made it impossible to accurately distinguish between these two types of muscles. This may imply that fungal effects on these muscles are unlikely to be cell specific at the time of biting. Our behavioral observations revealed that the mandibles worked normally in the hours preceding the death grip as infected ants were observed to self groom, cleaning their antennae and legs, which involves precise opening and closing of the mandibles as these appendages are pulled across the maxillae to be cleaned.

**Figure 4:**
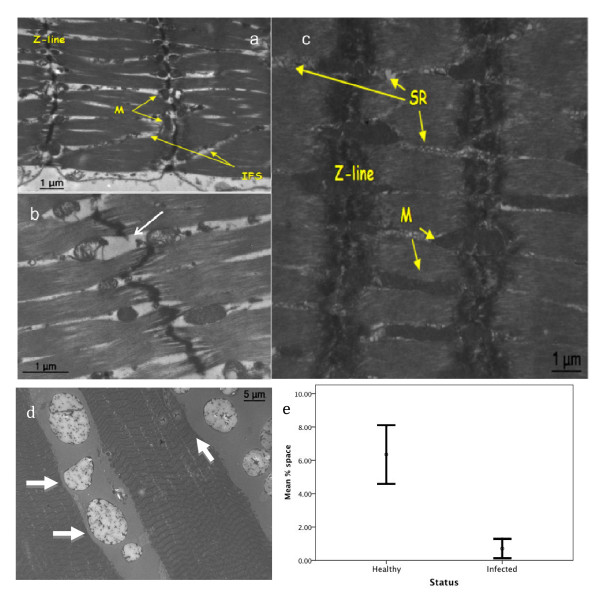
**Muscular atrophy in *O. unilateralis s.l *infected ants**. A) and B) are transmission electron micrographs (TEM) of infected ant mandible muscles. C) TEM shows uninfected muscles. The interfibrillar spaces (IFS) and reduced mitochondria (M) are evident in A & B. The z-line is thinned in A) and in B) an example of a broken z-line is shown by the white arrow. C) shows healthy muscle with prominent sarcoplasmic reticula (SR) and mitochondria. D) is a TEM of the mandible muscle of an *O. unilateralis *infected ant. The fibers are stretched and tearing is evident as lines running perpendicular to the sarcomeres. The large irregular structures between the muscle fibers are fungal cells. Note the impressions (white arrow) formed where they meet the muscle. The internal structures of the fungal cells have not been preserved using our method and the large white area within these cells reflects that and is therefore an artefact of fixing. E) Quantification of atrophy (mean (+/- SE) percent of interfibrillar space in the muscles cells) in healthy and infected ants. The difference is significant (see text).

At the sub-cellular level (as seen with TEM) the muscles of infected ants were very distinct from those of healthy individuals (Figure 4). Striated muscles (such as the mandible muscles) are composed of fibers that are multinucleated cells formed as a result of cell fusion. These fibers contain thick (myosin) and thin (actin) filaments which attach during cross-bridge cycling leading to muscle shortening. To achieve contraction mitochondria and sarcoplasmic reticulum provide energy (ATP) and ionic calcium (Ca^++^), respectively. At the end of each sarcomere unit there is a z-line (sarcomeres are in fact defined as the area between z-lines), which can be thought of as the anchor points for muscle contraction. Infected ants sampled during the death grip had broken z-lines and significantly less dense sarcoplasmic reticulum and mitochondria. This was determined from a measurement of the increase of interfibrillar spaces that appears following the loss of organelles, which in this case are sarcoplasmic reticulum and mitochondria (Kruskal-Wallis test, 20.25, df = 1, p < 0.0001, n = 6, Figure 4e). Similar to the light micrographs, the transmission electron micrographs showed a distinct atrophy in the muscles of infected ants.

**Figure 5:**
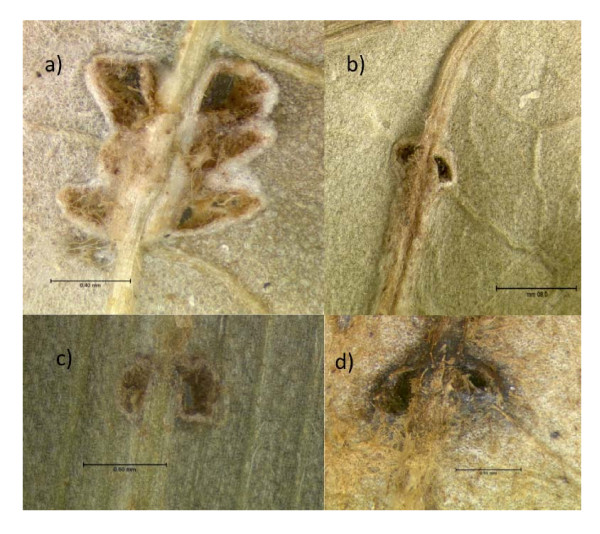
**Damage to plant tissue by zombie ants**. Photographs of abaxial vein and leaf lamina where zombie ants bit leading to scar formation by the plant. In A) two large holes are visible where the mandibles penetrated the lamina. In addition smaller marks are evident that formed as a result of rasping before the final death grip (see text). In B-D only one set of mandible marks occur. In all cases the vein has been cut. The arrows bars are 0.4 mm, 0.8 mm, 0.6 mm and 0.5 mm for A,B,C,D respectively.

Despite the apparent atrophy of muscles the behaviorally manipulated ants were able to exert considerable force. We removed 29 dead ants from diverse species of monocotyledonous and dicotyledonous leaves collected at our site (n = 10 and 19, respectively). On each leaf large puncture wounds were evident where the ant mandible had penetrated into the leaf (Figure 5). Only in two cases was the major vein on which biting was centered not cut into.

## Discussion

The first biologist documented to have seen *Ophiocordyceps*-induced body snatching extended phenotypes was Alfred Russell Wallace in 1859, as this features in his travelling notes from Sulawesi [30]. Yet despite the prestige of this original collector and the long period of time since that discovery, we have learned remarkably little about how these parasitic fungi might control the behavior of their insect hosts [1]. The present study and its recent predecessors [20,23] thus merely reveal fragments of a fascinating parasite adaptation to the host which coming decades may be able to resolve because of the revolutionary developments in sequenced based technology. We believe, however, that such studies will be particularly rewarding when they are based on field observations, the implications of which we summarize in the sections below.

### Multiple behavioral mechanisms explain niche choice by the fungus

Our first hypothesis was that the pre-biting behavior was important for positioning zombie ants in the precise niche observed during earlier studies at our field site [20,23]. We observed that infected ants could be discovered walking singly in the understory vegetation in the hours preceding biting and that their behavior was very distinct from that of healthy ants. Zombie ants regularly fell from the vegetation as they walked due to repeated convulsions (the whole body would shake, Figure 1), whereas the healthy ants that we observed all moved upwards toward the canopy without any such convulsive behavior. We suggest that the frequent falling is fungus-induced and functions to ensure that infected ants, which nest *ca*. 20 m above the forest floor, remain in the understory to ultimately attach themselves to leaves in the narrow zone of *ca*. 25 cm above ground [20].

Our data do not support the alternative explanation that zombie ants somehow measure the distance (number of steps for example) from the ground to the leaves on which they die. Likewise, we found no evidence to support the suggestion that manipulated ants assess leaf quality because they passed over so many leaves (99 on average) in such a short period of time that it is difficult to understand what quality would be assessed. Further, the falling and convulsing was continuing during this random walking making leaf quality comparison explanations unlikely.

While our behavioral observations support an extended phenotype explanation serving the *Ophiocordyceps *interests, they do not explain why infected ants occur on leaves of a distinct NNW orientation [20] or how the fungus causes its hosts to choose distinct parts of the leaf. Although ants were apparently manipulated into biting a wide range of plant species including both monocots and dicots (Figure 5), the location of the bites on main leaf veins remained highly invariant, with 98% of ants attaching to a major primary or secondary vein [20]. Our present work showed that the mandibles directly enter the vein of the leaf that is bitten (Figure 5) and the transition from walking to biting is abrupt and happens in a matter of minutes (*ca*. 20 h of observations following 16 zombie ants). Only in a few cases did the ants rasp the leaf or bite multiple times leading to multiple scars (Figure 5a). Our data does not explain why bites are centered on major veins or indeed what cues are used in deciding between lamina and vein. We suggest that the local topology (veins are raised above the lamina) provide a stimulus to biting behavior.

### Towards understanding the proximate mechanisms of the death grip

Our second hypothesis was that the death grip requires changes in the muscles of the mandible. We showed that there was considerable atrophy in the muscles of death grip ants, using both light microscopy (Figure 3) and sub-cellular transmission microscopy (Figure 4a-d). In the latter analysis we found a significant reduction in density of mitochondria and sarcoplasmic reticulum (Figure 4e). The changes that we observed suggest that muscles of zombie ants are lacking in energy for opening/closing work and in calcium ions for actin-myosin cross-bridge linking. Also, since the anchor points for actin-myosin cross linking (the Z-lines) were irregular or broken (Figure 4b) normal opening and closing would probably have become impossible. Once the ant has become attached, the muscles apparently atrophy rapidly inducing the characteristic lock-jaw followed by the death of the host ant some six hours later (Video 1). Thus while energy and ions for normal functioning are not present the atrophy likely functions to imbed the mandibles deep into the plant tissue due to this atrophy.

Whatever the time course of muscular degradation and its precise relationship to the extended phenotype (zombie behavior and biting) and unknown metabolite production, it is difficult not to expect that the enormous population of fungal cells in the head of zombie ants plays a decisive role (Figure 1). But much of this awaits clarification in future studies.

### Convergent evolution of zombie ant phenotypes

The synchronization of biting around solar noon that we found adds an intriguing angle to our study suggesting that future studies addressing the neurobiological and molecular mechanisms behind such possible adaptations will be highly rewarding. The sequences of behaviors we observed leading from walking to convulsions and finally to biting would make timed transcriptional profiles interesting and feasible. Fungi are well known to have clock genes useful in synchronizing activity. Further insights could be gained by extending such work to cover the other known cases where parasites have realized similar manipulative syndromes to create zombie ants.

Where *Ophiocordyceps *belongs to the fungal sub-phylum Ascomycota, there is also an entomopthoralean fungus *Pandora *that is known to turn European *Formica *wood ants into zombies at distinct times of the day [31]. While the details differ in that *Pandora *zombies do not produce fungal fruiting bodies, but sporulate directly from mycelium on the surface of the dead ants, it is striking that this convergent evolution happened in spite of the last common ancestor of these fungal clades having lived more than 500 mya [32]. Even more taxonomically distinct from both these fungal parasites is the wood ant brain worm, the trematode *Dicrocoelium dendriticum*, that also causes worker ants to leave their nest and bite into grass blades at distinct times of the day [33] to facilitate transmission to sheep as additional hosts (the ants acquire these trematode infections from a third host, a snail). With transcriptomic studies becoming more feasible, it would be highly intriguing to find out whether these extended phenotypes rely on similar or different secondary metabolites to express convergent behavior of infected hosts.

## Conclusion

Extended phenotypes in ants induced by fungal infections are a complex example of behavioral manipulation requiring coordinated changes of host behavior and morphology. Here we demonstrated some of the mechanisms by which such changes can be induced due to the effect of the fungi on ant walking behavior and muscular activity, leading to the pronounced biting that is a hallmark of this system. The insights from behavioral and histological studies point the way to more detailed work which can, ideally, lead to a detailed picture of the mechanisms by which organisms have evolved to control the behavior of other groups, even when they are in different kingdoms.

## Methods

Fieldwork took place in September 2007, *ca*. 20 km east of Trang in southern Thailand (7°32'49.50"N, 99°47'14.73"E). Here a 24 ha Forest Dynamics Plot (FDP) was established on the North-North-eastern slopes of the hills in the peninsular Khao Chong Botanic Garden as part of the Center for Tropical Forest Science (CTFS) pan-global FDP initiative http://www.ctfs.org. The site is exceptionally sandy with coarse and fine-grained sands and covered by a primary mixed evergreen forest, with an understory dominated by saplings (<1 m). The climate is tropical with seasonal monsoons and a mean monthly maximum temperature range from 29.0°C to 33.4°C, peaking in March-April. Rainfall is heaviest from May to September while the driest season is from November to February.

Live *Camponotus leonardi *ants were discovered by searching the understory vegetation for lone individuals. In previous studies the main host for *O. unilateralis *was *C. leonardi *and workers of this ant were encountered foraging in trails in the high canopy (>20 m) but these trails were rare in the understory [20,23]. Therefore, individual *C. leonardi *worker ants walking alone in the understory were all considered to be potentially infected justifying detailed observation as focal individuals (see also below). Because our initial indicator of infection was behavior, infection status of each ant was later checked. This was done in two ways: The first was by removing the ant from the leaf and dissecting it to allow a microscopic determination of fungal cells (hyphal bodies) inside the head capsule [20]. This is a reliable technique as hyphal bodies are clearly visible upon dissection [18]. The second technique was to return the following day to the marked ant that had fixed itself to a leaf to determine whether *O. unilateralis *had started growing from the body of the dead ant. This parasite fungus has a characteristic growth pattern following host death and a distinct colour (brownish hyphae) that facilitate a clear and unambiguous identification. All focal ants turned out to be infected.

During the focal individual observations, ants were monitored continuously from when they were discovered until 15 minutes after they had bitten a leaf. At each 5-minute interval the state of behavior was recorded (e.g. walking, grooming, resting or feeding) and non-state behaviors (events: falling, convulsing, rasping leaves and biting leaves) were recorded as they occurred. The number of leaves that ants traversed was recorded as well. This only happened for 8 individuals because accurately counting the number of leaves that an ant traversed over the entire monitoring period (around 2 hours) was difficult because leaves of small understory plants often overlap which hampered accurate counting.

Ants from a foraging trail on a liana where assumed to be uninfected since they displayed normal behavior (foraging, communication with other ants on the trail and a display of aggression when disturbed). Individuals to be used for focal observations of healthy ants were removed from the trail and placed directly onto the forest floor. Also these ants were continuously monitored by one of three observers in the same way as described above for the infected ants. The observation period stopped when the ant ascended a tree to a height of *ca*. 1.5 m (where they were recollected) or if the ant was lost or predated upon. None of the infected ants were lost or predated upon, so this category was not mentioned above.

Searches for infected ants at other times of the day besides 09:00-14:00 hrs occurred regularly through the course of fieldwork in 2006 (September/October 35 days; November, 3 days) and 2007 (January, 27 days; September, 25 days). In September 2006 video recording was used on ants that were discovered after they had bitten leaves. The leaf on which an ant was biting was recorded for 10 minutes and behavior subsequently scored.

We used the freely available Solar Position Calculator to obtain the solar time and solar altitude for each ant that bit into a leaf. Ant observations took place on 10, 11, 12 13, 14, 15, 16 and 19 September 2007. As an example, sunrise was at 6:15 on the 10^th ^and sunset was at 18:21 on the 10^th ^of September. We used Latitude 7.54347 and Longitude 99.798 to calculate the solar time and elevation.

### Histology

The heads of healthy ants and ants that had bitten leaves were removed and were fixed in 2% glutaraldehyde (buffered at pH 7.3 with 50 mM sodium cacodylate and 150 mM saccharose) and post-fixed in 2% osmium tetroxide in the same buffer. Dehydration was carried out in a graded acetone series and preceded embedding in Araldite and sectioning with a Reichert Ultracut E microtome. Thin sections of 1 μm for light microscopy were stained with methylene blue and thionin. Ultrathin sections of 70 nm were double stained (lead citrate and uranyl acetate) and examined with a Zeiss EM900 transmission electron microscope.

### Taxonomic note

We use the designation *sensu lato *throughout to emphasize that based on an on-going taxonomic revision of the globally dispersed fungal species *Ophiocordyceps unilateralis*, a new name will be required for the species at our study site [34].

## Authors' contributions

Conceived and designed the experiments: DPH. Performed the experiments: DPH, SBA, JB, WH Analyzed the data: DPH, SBA, JB Wrote the paper: DPH, JJB, JB, SBA, NLH-J, MBP, JJK. All authors read and approved the final manuscript.

## Supplementary Material

Additional file 1 **Trails of the ant *Camponotus leonardi***. The video shows ants running in a trail on a branch above the forest floor in a tropical forest in Southern Thailand. Click here for file

Additional file 2 **A zombie ant biting a leaf vein**. An ant attached by its mandibles to the main vein of a leaf in a tropical forest in Southern Thailand. The ant remains attached until its death and does not respond to external factors such as another ant approaching as in the video. Click here for file

## References

[B1] Moore J Parasites and the behavior of animals 2002 Oxford: Oxford University Press

[B2] Lafferty K Kuris AM Parasitic castration: the evolution and ecology of body snatchers Trends in Parasitology 2009 25 12 564 572 10.1016/j.pt.2009.09.003 19800291

[B3] Poulin R Progenesis and reduced virulence as an alternative transmission strategy in a parasitic trematode Parasitology 2001 123 623 630 11814049 10.1017/s0031182001008794

[B4] Lefevre T Adamo SA Biron DG Misse D Hughes D Thomas F Invasion of the Body Snatchers: the diversity and evolution of manipulative strategies in host-parasite interactions Advances in Parasitology 2009 68 45 83 19289190 10.1016/S0065-308X(08)00603-9

[B5] Dawkins R The extended phenotype 1982 Oxford: W.H. Freeman

[B6] Rogers ME Bates PE *Leishmania *manipulation of sand fly feeding behavior results in enhanced transmission Plos Pathogens 2007 3 6 e91 10.1371/journal.ppat.0030091 17604451 PMC1904410

[B7] Eberhard WG Spider manipulation by a wasp larva Nature 2000 406 6793 255 256 10.1038/35018636 10917517

[B8] Kuris AM Hechinger RF Shaw JC Whitney KL Aguirre-Macedo L Boch CA Dobson AP Dunham EJ Fredensborg BL Huspeni TC Ecosystem energetic implications of parasite and free-living biomass in three estuaries Nature 2008 454 7203 515 518 10.1038/nature06970 18650923

[B9] Thomas F Adamo S Moore J Parasitic manipulation: where are we and where should we go? Behavioural Processes 2005 68 3 185 199 10.1016/j.beproc.2004.06.010 15792688

[B10] Hughes DP Kronauer DJC Boomsma JJ Extended Phenotype: Nematodes turn ants into bird-dispersed fruits Current Biology 2008 R294 295 10.1016/j.cub.2008.02.001 18397736

[B11] Poulin R The evolution of parasite manipulation of host behavior: a theoretical analysis Parasitology 1994 109 S109 S118 10.1017/S0031182000085127 7854845

[B12] Poulin R Manipulation of host behaviour by parasites: a weakening paradigm? Proceedings of the Royal Society of London Series B 2000 267 1445 787 792 10.1098/rspb.2000.1072 10819148 PMC1690597

[B13] Dawkins R Parasites, desiderata lists and the paradox of the organism Parasitology 1990 100 S63 S73 10.1017/S0031182000073029 2235064

[B14] Sanchez MI Ponton F Schmidt-Rhaesa A Hughes DP Misse D Thomas F Two steps to suicide in crickets harbouring hairworms Animal Behaviour 2008 76 1621 1624 10.1016/j.anbehav.2008.07.018

[B15] Evans HC Entomogenous fungi in the tropical forest ecosystems: an appraisal Ecological Entomology 1982 7 47 60 10.1111/j.1365-2311.1982.tb00643.x

[B16] Evans HC Samson RA *Cordyceps *species and their anamorphs pathogenic on ants (Formicidae) in tropical forest ecosystems. II. The *Camponotus *(Formicinae) complex Transactions of the British Mycolocical Society 1984 82 127 150 10.1016/S0007-1536(84)80219-3

[B17] Van Pelt A The occurrence of a *Cordyceps *on the ant *Camponotus pennsylvanicus *(De Geer) in the Highlands, N.C. region Journal of the Tennesee Academy of Sciences 1958 33 120-122

[B18] Hughes DP Evans HC Hywel-Jones NL Boomsma JJ Armitage SAO Novel fungal disease in complex leaf-cutting ant societies Ecological Entomology 2009 34 2 214 220 10.1111/j.1365-2311.2008.01066.x

[B19] Hölldobler B Wilson EO The ants 1990 Cambridge, Mass.: Harvard University Press

[B20] Andersen SB Gerritsma S Yusah KM Mayntz D Hywel-Jones NL Billen J Boomsma JJ Hughes DP The life of a dead ant: the expression of an adaptive extended phenotype American Naturalist 2009 174 3 424 433 10.1086/603640 19627240

[B21] Kuris AM Trophic interactions: similarity of parasitic castrators to parasitoids Quarterly Review of Biology 1974 49 129 148 10.1086/408018

[B22] Evans HC Samson RA *Cordyceps *species and their anamorph pathogenic on ants (Formicidae) in tropical forest ecosystems. I. The *Cephalotes *(Myrmicinae) complex Transactions of the British Mycolocical Society 1982 79 431 453 10.1016/S0007-1536(82)80037-5

[B23] Pontoppidan M-B Himaman W Hywel-Jones NL Boomsma JJ Hughes DP Graveyards on the move: the spatio-temporal distribution of dead *Ophiocordyceps*-infected ants PLoS ONE 2009 4 3 e4835 10.1371/journal.pone.0004835 19279680 PMC2652714

[B24] Sanjuán T Guillermo Henao L Amat G Distribución espacial de *Cordyceps *spp. (Ascomycotina: Clavicipitaceae) y su impacto sobre las hormigas en selvas del piedemonte amazónico de Colombia Revista de Biologia Tropical 2001 49 3-4 945 955 12189826

[B25] Evans HC Natural control of Arthropods with special reference to ants (Formicidae) by fungi in the tropical high forest of Ghana The Journal of Applied Ecology 1974 11 1 37 49 10.2307/2402003

[B26] Hughes DP Parasitic manipulation: a social context Behavioural Processes 2005 3 263 266 10.1016/j.beproc.2004.09.006 15792704

[B27] Hughes DP d'Ettorre P, Hughes DP The extended phenotype within the colony and how it obscures social communication Sociobiology of Communication: an interdisciplinary perspective 2008 Oxford Oxford University Press

[B28] Hughes DP Pierce NE Boomsma JJ Social insect symbionts: evolution in homeostatic fortresses Trends in Ecology & Evolution 2008 23 12 672 677 10.1016/j.tree.2008.07.011 18951653

[B29] Pearson K The problem of the random walk Nature 1905 72 342 342

[B30] Fawcett W Description of *Cordyceps llyodii *in ants Annals and Magazine of Natural History 1886 5 XVIII 317

[B31] Marikovsky PI On some features of behaviour of the ants *Formica rufa *L. infected with fungous disease Insectes Sociaux 1962 9 173-179

[B32] Blair JE Fungi 2009 Oxford Oxford University Press

[B33] Manga-Gonzalez MY Gonzalez-Lanza C Cabanas E Campo R Contributions to and review of dicrocoeliosis, with special reference to the intermediate hosts of *Dicrocoelium dendriticum* Parasitology 2001 123 S91 S114 11769295 10.1017/s0031182001008204

[B34] Evans HC Elliot SL Hughes DP Hidden diversity behind the Zombie-ant fungus *Ophiocordyceps unilateralis*: Four new species described from Carpenter ants in Minas Gerais, Brazil PLoS ONE 2011 6 3 e17024 10.1371/journal.pone.0017024 21399679 PMC3047535

